# Role of Sirolimus in renal tubular apoptosis in response to unilateral ureteral obstruction: Erratum

**DOI:** 10.7150/ijms.71282

**Published:** 2022-03-03

**Authors:** Mei Yang, Yang-yang Zhuang, Wei-wei Wang, Hai-ping Zhu, Yan-jie Zhang, Sao-ling Zheng, Yi-Rrong Yang, Bi-Cheng Chen, Peng Xia, Yan Zhang

**Affiliations:** 1 Department of Intensive Care Unit, the First Affiliated Hospital of Wenzhou Medical University, Wenzhou, Zhejiang Province, China 325015; 2 Transplantation centre, the First Affiliated Hospital of Wenzhou Medical University, Wenzhou, Zhejiang Province, China 325015; 3 Zhejiang Provincial Top Key Discipline in Surgery, Wenzhou Key Laboratory of Surgery, Department of Surgery, The First Affiliated Hospital of Wenzhou Medical University, Wenzhou, Zhejiang Province 325015, China

In our paper 1, Figure 1 and Figure 2 should be corrected as follows.

## Figures and Tables

**Figure 1:**
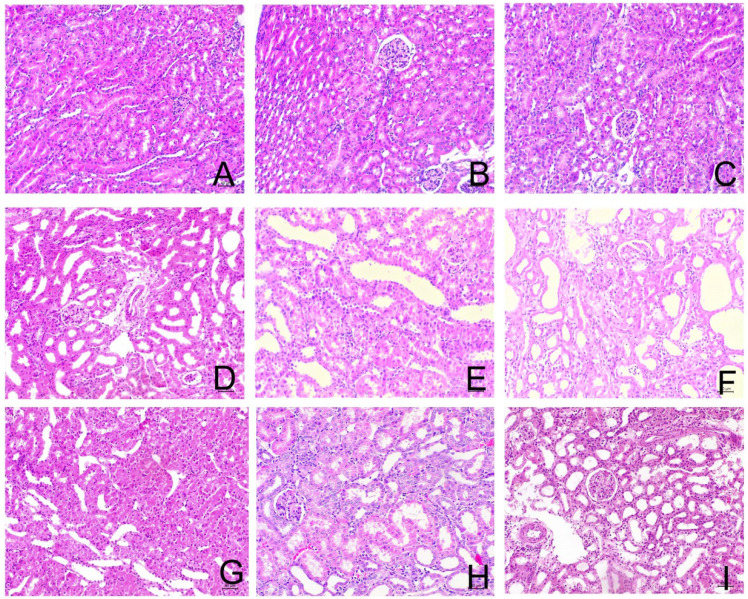
Sirolimus attenuated the histological changes in the obstructed kidney induced by UUO. Representative hematoxylin-eosin staining micrographs of (A, B, C) Sham group, (D, E, F) UUO group and (G, H, I) Sirolimus group. Original magnification×200.

**Figure 2:**
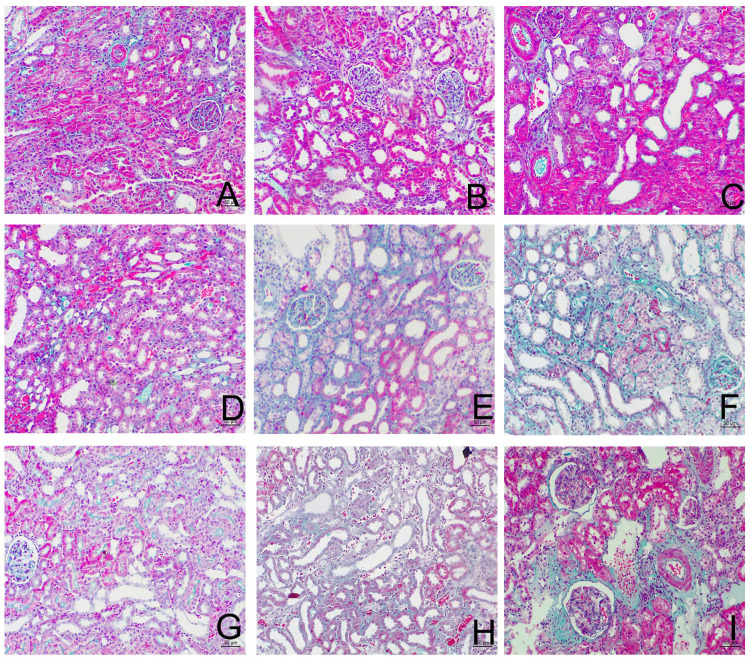
Sirolimus attenuated the interstitial collagen deposition in the obstructed kidney induced by UUO. Representative Masson's trichrome staining micrographs of (A, B, C) Sham group, (D, E, F) UUO group and (G, H, I) Sirolimus group. Original magnification×200.
